# Relationship between Higher Atherogenic Index of Plasma and Oxidative Stress of a Group of Patients Living with Sickle Cell Anemia in Cameroon

**DOI:** 10.1155/2020/9864371

**Published:** 2020-03-17

**Authors:** Landry Nguepnkep Kubong, Prosper Cabral Nya Biapa, Bernard Chetcha, Nicolas Yanou-Njintang, Vicky Jocelyne Moor Ama, Constant Anatole Pieme

**Affiliations:** ^1^Faculty of Sciences, University of Ngaoundere, Ngaoundere, Cameroon; ^2^Faculty of Sciences, University of Dschang, Dschang, Cameroon; ^3^Faculty of Medicine and Biomedical Sciences, University of Yaoundé I, Yaounde, Cameroon

## Abstract

Dyslipidemia is highly prevalent in sickle cell anemia (SCA) patients and is one of the major risk factors for cardiovascular diseases induced by oxidative stress in Africa. The aim of this research was to investigate the correlation between higher atherogenic index of plasma (API) and oxidative stress in a group of patients living with SCA in Cameroon. *Methods*. A group of 85 homozygote SS patients (male and female) were enrolled at the Central hospital of Yaounde in Cameroon between May and October 2017. After informed consent through the signature of a consent form was obtained, the plasma was collected to determine the lipid profile while the lysate solution of RBC was used to explore some markers of oxidative stress using spectrophotometric methods. *Results*. Among the 85 patients included in our study, the mean age was 30 ± 5 years and the female to male ratio was 0.97. The majority of the patients (52–81%) had dyslipidaemia, and 22.4% of the patients demonstrated a higher level of atherogenic index of plasma. The patients with a higher level of total cholesterol (TC) (>240 mg/dl) and low-density lipoprotein (LDL-C) (>159 mg/dl) had at least 1,334 fold of malondialdeheyde (MDA) concentration than those with normal level. Also in the same patients, the higher atherogenic plasmatic index (API) significantly (*p* < 0.05) increased with the concentration of MDA. Except HDL-C, the other parameters of lipid profile had significant (*p* < 0.05) correlation with reduced glutathione (GsH) and total antioxidant capacity (TAC). The significant (*p* < 0.05) and linear regression was found between the increased MDA and higher API. *Conclusion*. Dyslipidemia increases oxidative stress and higher API which leads to coronary vascular disease in patients with SCA.

## 1. Background

Sickle cell anemia (SCA) is a genetic disease with autosomal recessive inheritance. It results from a point mutation of adenine by thymine (GAG/GTG) at the sixth codon of the *β*-globin chain on chromosome 11. This mutation results in the substitution of glutamic acid by valine (Glu/Val) which leads to the synthesis of abnormal hemoglobin (S) [1]. It is the most common genetic disease in the world and the most prevalent in sub-Saharan Africa, with about one case for every 350 births [2]. In Africa, the average survival of children with sickle cell disease is less than five years, and it is estimated that approximately 50% to 80% of the 400,000 children born each year in Africa with sickle cell disease die before the age of five [3]. In Cameroon, the prevalence is 2 to 3% [4, 5]. In homozygous sickle cell disease, studies have shown disruption of serum lipid and lipoprotein metabolism [6]. Among the various physiological aspects of sickle cell disease, accelerated auto-oxidation and tissue oxygenation disorders are responsible for the radical reaction and its complications [7]. The production of free radicals leads to lipoperoxidation, a sequence of major events that contribute to the reduction of the half-life of red blood cells [8]. HbS is reported to have an accelerated autoxidation rate, with increased generation of reactive oxygen species (ROS) in sickled RBCs. ROS production was found to be 10–30 fold higher in the RBCs, platelets, and polymorphonuclear neutrophils of patients with SCA than in normal subjects [9]. Previous results reported that the level of antioxidant markers, superoxide dismutase (SOD), total antioxidant capacity (TAC), and catalase (CAT) was higher in healthy patients compared to SDC patients in Cameroon [5]. The sickle erythrocytes generate approximately two times more amounts than usual amount of superoxide, peroxide, and hydroxyl radicals which can react with nitrogen oxide (NO) converting it into more potent reactive species which may damage the cell membrane [10]. Autoperoxidation of polyunsaturated fatty acids (PUFAs) is also initiated and increases free radicals, and one of the end products of this oxidation is malondialdehyde. Lipid abnormalities and increased oxidative stress may accelerate the process of atherosclerosis in patients suffering from SCA. This study evaluates the association of oxidative stress and atherogenic index of plasma so as to assess the cardiovascular risk in sickle cell anemia patients.

## 2. Methods

### 2.1. Recruitment of Participants

We conducted a cross-sectional study between May and October 2017 at the Central Hospital of Yaounde which is a secondary hospital in the town. A total of eighty-five sickle cell patients (43 males and 42 women) were enrolled in the study. The patients were consecutively recruited at the same hospital, and their blood samples were analyzed in the Department of Biochemistry of the Faculty of Medicine and Biomedical Sciences of the University of Yaounde I, Cameroon. The sample size was calculated using the formula of Lorentz which takes into consideration the prevalence of homozygous SS patients in Cameroon. (2-3%) [3]. None of the patients enrolled had a history of concomitant chronic illness such as diabetes, evidence of cardiovascular, hepatic, renal, or gastrointestinal diseases, or the presence of chronic infectious illnesses (rheumatoid arthritis and AIDS). Homozygous sickle cell (SS) patients, with males and females above 15 years old, who were known and regularly monitored in the hospital and who accepted to participate in the study were enrolled. Information on the study was given to the potential participants and their legal guardians. Patients or their guardians read and signed the informed consent form. For patients unable to read the consent form, they were helped by their parents or guardians. For each participant, demographic data were obtained and noted on a prestructured data collection form. The study was approved by the ethical committee of the Faculty of Medicine and Biomedical Sciences of the University of Yaounde 1 (Cameroon) and ethics committee for human health research of the regional delegation of the center region under the reference number CE No 00485/CREHHS/2017. All patients with sickle cell anemia and other pathologies such as AIDS and diabetes or those taking medication that may influence oxidative status such as vitamins A or E except folic acid were excluded from this study.

### 2.2. Blood Collection

Five milliliters of the venous blood were collected from each participant into a tube containing ethylenediaminetetraacetate (EDTA) after at least 12h fasting. The blood was then centrifuged at 4000 rpm for 15 min. The plasma and buffy coat were removed, sickle RBCs were lysed with ice cold water, and the clear lysate was obtained after spinning down the cell debris at 5000 rpm for 10 min at 4°C.

## 3. Biochemical Analyses

### 3.1. Antioxidant Enzyme Activities

The solution obtained from lysed red blood cells was used to investigate the markers of oxidative stress. The superoxide dismutase (SOD) activity was determined using the Misra and Fridovich method which is because SOD inhibits the oxidation of adrenaline to adrenochrome. Adrenochrome formed was detected in a spectrophotometer at 480 nm by its pink color [11]. The catalase activity was determined following the addition of hydrogen peroxide and dichromate/acetic acid by the detection of perchloric acid precipitates whose absorbance was read at 570 nm by spectrophotometry [12]. The catalase activity was expressed as the micromoles of H2O2/min/mg of protein. Glutathione peroxidase activity was determined by the peroxidase kit (CAS Number 7722-84-1, Sigma Aldrich). It is an enzymatic method using pyrogallol as the substrate to determine the activity of glutathione peroxidase, and the absorbance was read at 420 nm. The total antioxidant capacity (TAC) of the lysis solution was measured using a ferric reducing antioxidant power (FRAP), a method that determined the capacity of a sample to reduce iron (Fe^3+^) at the acidic pH (3.6). An intense blue color was formed when the ferric tripyridyltriazine (TPTZ-Fe^3+^) complex was reduced to ferrous tripyridyltriazine (TPTZ-Fe^2+^) and the absorbance measured at 593 nm. The result was expressed in micromolar [13]. Lipid peroxidation assay was performed by a formerly described protocol [14]. Aldehydes, lipid peroxidation of products, and malondialdehyde (MDA), an end product of the lipid peroxidation of erythrocytes, reacted with thiobarbituric acid (TBA) to form a colored complex called thiobarbituric acid reactive substance (TBARS), which was assayed spectrophotometricaly at 532 nm and expressed in micromoles.

### 3.2. Determination of the Lipid Profile

Plasma total cholesterol (TC) and triglycerides (TG) and high-density lipoprotein cholesterol (HDL-C) concentrations were determined by enzymatic colorimetric assays (Kits Cypress, Belgium), and low-density lipoprotein cholesterol (LDL-C) was determined by calculation using the formula of Friedewald: VLDL-C = TG/2.2. The atherogenic index of plasma (AIP) was calculated as the logarithmically transformed ratio of molar concentrations of TG to HDL-C [15].

### 3.3. Statistical Analysis

The normality of our main variables was examined in order to estimate the type of distribution [16]. At the end of this estimate, parametric tests were chosen. The results were expressed as mean ± standard deviation. The chi-square test was used to compare the frequencies, and the independent *t*-test and ANOVA helped to compare means. The Pearson correlation allowed estimating the level of association between quantitative variables. Data were prepared in the Excel software and analyzed with the SPSS software (Statistical Package for Social Sciences) version 16.0. A value *p* < 0.05 was considered to be significant.

## 4. Results

### 4.1. Sociodemographic Parameters

We recruited 85 sickle cell patients of both sexes (women 49.4% and men 50.6%) representing a ratio of 0.97. The mean age was 30 ± 5 years, and the most represented group belonged to patients between 20 and 29 years (Table 1). The distribution of sample population according to the reference value of the lipid profile is given in Table 2. According to these results, except for the TG, the majority (51.8–82.4%) of the sample population had an abnormal level of HDL-C, LDL-C, and TC demonstrating that they can have dyslipidaemia among SS sickle population. In general, abnormal values were significantly (*p* < 0.0001) elevated compared to normal for all lipid profile parameters except for triglycerides. Only 22.4 % SCA patients demonstrated dyslipidemia with higher atherogenic plasmatic index (API).

#### 4.1.1. Effect of Lipid Profile on Some Markers of Oxidative Stress

The effect of lipid profile on some markers of oxidative stress is presented in Table 3. This result shows that the level of lipid profile did not affect SOD and GPx activities. The group of patients with normal level of total cholesterol had the level of FRAP significantly higher (1.14 fold) than the abnormal group, GsH (1.211 fold), and CAT (2 fold). The same findings were noted with FRAP where the concentration was 1.37 fold and 0.87 fold higher, respectively, for TG and LDL-C. In contrary, patients with abnormal level of TC and HDL-C had at least 1.334 fold higher concentrations of MDA compared to those who had normal level. The results of the effect of atherogenic plasmatic index on markers of oxidative stress are presented in Table 4. In general, when the plasmatic atherogenic index increases, the level of MDA also significantly increases (*p* < 0.05) while the activity of SOD significantly reduces (*p* < 0.05). The variation of API did not significantly affect the other markers of oxidative stress such as GPx, GsH, total antioxidant capacity, and catalase (Table 4).

### 4.2. Effects of Some Extrinsic Factors on Some Markers of Oxidative Stress and Folic Acid Intake

Table 5 shows the influence of gender and the time of sickle cell anemia detection on oxidative stress parameters. In general, only women have significant (*p* < 0.05) lower concentration of total antioxidant capacity compared to men. Patients in whom SCA was detected in less than one year demonstrated a significant lower concentration of MDA compared to those in which SCA was found after one year. The gender and the period of SCA detection did not significantly affect the other markers of oxidative stress (Table 5). The influence of folic acid intake, some markers of oxidative stress, and lipid profile is presented in Table 6. The results show that the folic acid intake significantly (*p* < 0.005) reduced the TG levels as well as that of MDA. The levels of others parameters either for lipid profile or oxidative stress were not significantly affected by the intake of folic acid. The cross tabulation (Table 7) presents a predicting influence of folic acid intake and hospitalization on the venue of crisis among SCA patients. The *p* value of 0.027 and 0.002 revealed a significant impact of folic acid intake and hospitalization, respectively, on the development of crisis. The relative risk of the sickle cell patients to develop crisis when they are hospitalized or consumed folic acid (0.775 and 0.429, respectively) is less than 1 demonstrating that these factors have beneficial or protective effects on the patients.

### 4.3. Correlation between Some Markers of Oxidative Stress, Lipid Profile, Atherogenic Plasmatic Index, and Prediction of Crisis to Sickle Cell Patients

In order to have an idea on the relation between lipid profile and markers of stress, a Pearson correlation was performed and presented in Table 8. The results show that except HDL-C, the other parameters of lipid profile have significant relationship with two markers of oxidative stress (GsH and FRAP). TC and LDL-C have significant negative correlation with GsH and FRAP, respectively, while TG demonstrated significant positive relation with them. The other significant correlations were found either among the markers of oxidative stress or lipid profile (Table 8).

**Figure 1:**
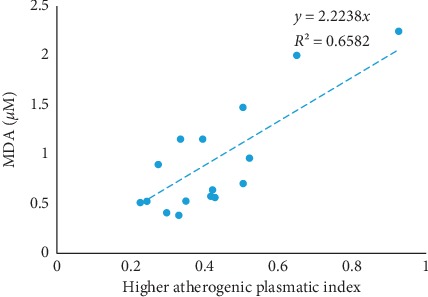
Linear regression between the MDA level and the high API.

In order to investigate the relationship between the markers of oxidative stress and lipid profile with the aim to predict the crisis among sickle cell patients, a series of statistical analysis with the Hosmer–Lemeshow tests were carried out and the results are presented in Table 9. The first result demonstrated that CAT and LDL can statistically predict the onset of the crisis in these patients. The results show that there is a positive relationship between LDL-C variable demonstrating that patients with a higher level of LDL-C may develop crisis. In contrary, those with a higher level of CAT had less crisis. The association between higher API and MDA was assessed using a linear regression. The result presented in Figure 1 shows that there is a linear and positive link that exist between MDA and API (*R*^2^ = 0.6582) demonstrating that the two markers increased or reduced simultaneously in the same manner.

## 5. Discussion

Our research aimed to investigate the relationships between dyslipidemia, oxidative markers, and painful crisis in patients living with SCA in Cameroon [16, 5]. Our sample population was made of almost equal number of male and female, and most of them had normal weight and routinely took folic acid. The most representative age was between 20 and 30 years, and they had an abnormal level of lipid profile except TG. The impact of disordered lipid metabolism on the course of SCA and its numerous complications are not yet clearly defined. Also, there is little information on the lipid profile of SCA risk of atherogenesis and oxidative stress. Several mechanisms contribute to produce high oxidative stress in sickle cell patients, including the excessive levels of cell-free hemoglobin with its catalytic action on oxidative reactions, the characteristic recurrent ischemia-reperfusion injury, a chronic proinflammatory state, and higher autoxidation of sickle hemoglobin (HbS) [17]. Abnormal lipid homeostasis has been reported in SCA as well as other hematological disorders. Our result demonstrated that SCA patients with normal level of TC significantly have a higher concentration of GsH, FRAP, and CAT. Similar results were found with TG and FRAP. In contrary, the level of MDA was higher in patients with a higher level of LDL-C. Similar results were noted by similar studies which attributed these increased lipid peroxidation observed in SCA patients to increase autoxidation and iron decompartmentalisation [5, 18]. During this process, the high metabolic turnover of polymerization and depolymerization upon deoxygenation and reoxygenation, respectively, is a potentially important source of ROS production [19] which can either increase lipid peroxidation or reduce the antioxidant defense of the organism. The level of lipid profile and oxidative stress of the SCA patients are influenced by many factors such as gender, folic acid intake, and period of detection of the sickle cell disease [20]. It is proposed that folate in anemia raises hemoglobin levels and helps provide a healthy reticulocyte response. Hence, in the management of hemolytic anemia in SCD, folic acid replenishes the depleted folate stores necessary for erythropoiesis [21]. Vitamins like folic acid are involved in the metabolism of several macromolecules. Folate is transported into cells by two distinct transport mechanisms. The first is a low-affinity system called reduced folate carrier 1 (RFC1). The second system is a high-affinity system that involves three folate receptors (FRs) expressed at the cell surface and internalized by receptor-mediated endocytosis. When folate levels are low, the membrane-bound folate receptors are used [22]. Previous studies showed that cholesterol regulates the rate of cellular folate import by facilitating the clustering of membrane-bound folate receptors on the cell membrane [23]. Our results showed that patients who received folic acid have a higher level of TC, HDL-C, LDL-C, and TG compared to those who did not take this vitamin. Other researchers indicated that folate deficiency has been shown to result in the accumulation of triacylglycerol (TG) in the liver. However, these results are different from other studies which indicated that the higher levels of folate were associated with a favorable lipoprotein profile independent of the covariables, age, and gender. Therefore, the higher level of lipid can be explained by the dosage of folic acid which is lower in these patients. In our study, the supplementation of patients with folate has nonsignificantly increased enzymatic and nonenzymatic antioxidant markers while significantly reducing the level lipid peroxidation by decreasing MDA concentration. These findings can be interpreted as a result of decreased production of free radicals during the lowering through several mechanisms. Folate can thus be considered as an effective antioxidant. The increased production of ROS in SCD can modify the response to a variety of pathophysiological conditions including inflammation, hypoxia, metabolism of drugs or alcohol, and deficiency in antioxidant enzymes. Reactive oxygen species can cause significant damage to biomolecules since membrane lipids readily react with ROS resulting in lipid peroxidation [24]. Our results demonstrated negative significant correlation between TG, LDL-C, HDL-C, and FRAP or GsH confirming that the accumulation of lipid will lead to the reduction of antioxidant defenses. Also, the linear regression between AIP and MDA that we obtain confirms that the increase of oxidative stress accelerates the process of cardiovascular complications in adult SCD patients. Our inability to find a higher level of dependency between AIP and MDA could perhaps find an explanation in the lack of power as the study was a cross-sectional one with a relative small sample size. Another limitation of our study is that there are no local standard values to evaluate the oxidative stress markers which would have given more input on our results.

## 6. Conclusion

The chronically elevated oxidative stress in SCD might play a significant role in the development of cardiovascular diseases through the increase of API. Secondary products of oxidative stress may potentially serve as the biomarkers of disease severity in SCA. The potential of antioxidants and agents with antioxidative effects for preventing or delaying the development of organ complications in sickle cell patients deserves thorough investigation.

## Figures and Tables

**Table 1:** Characteristics of the population of the study.

Characteristics	Percentage (%)	
Gender	Female	49.4
	Male	50.6

Body mass index (BMI)	Underweight	36.5
	Normal	62.4
	Overweight	1.2

Alcohol consumption	No	76.6
	Yes	22.4

Folic acid consumption	No	20
	Yes	80

Hydroxyurea consumption	No	95,3
	Yes	4.7

Crisis	No	51.8
	Yes	48.2

Age interval	< 20 years	42.2
	[20–29] years	44.7
	>30 years	12.9

**Table 2:** Distribution of population according to the normal level of lipid profile and percentage of lipid profile.

Lipid profile		Percentage (%)	*p* value	N
TC (mg/dl)	Normal (<200 mg/dl)	48.2	0.000	85
	Abnormal	51.8		

TG (mg/dl)	Normal (< 135 mg/dl)	77.6	0.000	85
	Abnormal	22.4		

LDL-C (mg/dl)	Normal (< 160 mg/dl)	32.9	0.000	85
	Abnormal	67.1		

HDL-C (mg/dl)	Normal (> 40 mg/dl)	17.6	0.000	85
	Abnormal	82.4		

AIP	Low (< 0.11)	60.0		
	Moderate (0.11–0.21)	17.6		
	High ( > 0.21)	22.4		

Chi-square test at *p* < 0.05. LDL-C = low-density lipoprotein, TC = total cholesterol, TRIG = triglyceride, and HDL-C = high-density lipoprotein. Reference values: TC between 140 and 250 mg/dl; TG not greater than 150 mg/dl; HDL-C between 50 and 60 mg/dl. AIP = atherogenic index of plasma.

**Table 3:** Effect of lipid profile on some markers of oxidative stress.

Lipid profile		SOD (UI/mg protein)	GsH (*µ*M)	GPx (UI/mg protein)	FRAP (*µ*M)	CAT (UI/mg protein)	MDA (*µ*M)
TC (mg/dl)	Normal	1.01 ± 1.00	4.62 ± 0.34^*∗*^	0.64 ± 1.03	272.12 ± 12.5^*∗*^	2.26 ± 0.55^*∗*^	0.97 ± 0.77
	Abnormal	0.78 ± 0.72	3.80 ± 1.34	0.52 ± 0.27	238.59 ± 10.5	1.10 ± 0.56	1.30 ± 0.2

TG (mg/dl)	Normal	0.95 ± 0.95	4.67 ± 0.2	0.59 ± 0.83	275.42 ± 16.47^*∗*^	1.14 ± 0.59	1.10 ± 0.17
	Abnormal	0.71 ± 0.48	4.10 ± 0.4	0.55 ± 0.26	200.34 ± 9.68	1.29 ± 0.42	2.15 ± 0.2

LDL-C (mg/dl)	Normal	0.82 ± 0.10	4.48 ± 0.28^*∗*^	0.74 ± 0.23	234.50 ± 71.91	1.26 ± 0.10	0.89 ± 0.16
	Abnormal	0.93 ± 0.13	3.71 ± 0.24	0.50 ± 0.03	266.48 ± 78.55	1.13 ± 0.07	1.26 ± 1.46

HDL-C (mg/dl)	Normal	0.90 ± 0.15	3.66 ± 0.32	0.53 ± 0.07	236.46 ± 54.87	1.18 ± 0.10	0.67 ± 0.14
	Abnormal	0.89 ± 0.11	4.35 ± 0.24	0.59 ± 0.09	260.12 ± 9.70	1.17 ± 0.07	1.24 ± 0.16^*∗*^

Results are expressed as mean ± SD. ^*∗*^indicates the values are statistically significant in the same column. *p* < 0.05. GsH = reduced glutathione, SOD = superoxide dismutase, FRAP = ferric reducing antioxidant power, GPx = glutathione peroxidase; LDL-C = low-density lipoprotein, TC = total cholesterol, TG = triglyceride, and HDL-C = high-density lipoprotein.

**Table 4:** Relation between atherogenic plasmatic index and markers of oxidative stress.

Atherogenic plasmatic index	MDA (*µ*M)	SOD (UI/mg protein)	GsH (*µ*M)	GPx (UI/mg protein)	FRAP (*µ*M)	CAT (UI/mg protein)
Low (*n* = =51)	0.02 ± 1.4^a^	1.39 ± 0.6^ab^	4.21 ± 2.1	0.60 ± 0.3	280.96 ± 7.3	1.33 ± 0.3
Moderate (*n* = =15)	1.21 ± 1.1 ^b^	0.85 ± 0.1^b^	3.54 ± 1.3	0.52 ± 0.1	263.36 ± 10.0	1.14 ± 0.6
High (*n* = =19)	1.46 ± 1.0 ^b^	0.63 ± 0.1^bc^	3.79 ± 1.8	0.56 ± 0.1	244.45 ± 7.0	1.11 ± 0.3

Results are expressed as mean ± SD. Values subscripted with different letters are significantly different in the same column ^*∗*^*p* < 0.05. GsH = reduced glutathione, SOD = superoxide dismutase, FRAP = ferric reducing antioxidant power, MDA = malondialdehyde, CAT = catalase, and GPx = glutathione peroxidase.

**Table 5:** Influence of gender and the age of sickle cell anemia detection on the stress profile.

	Gender	N	Mean ± SD	Duration SCA	N	Mean ± SD
MDA (*μ*M)	Men	43	1.34 ± 1.63	≤1 year	23	1.61 ± 2.02
	Women	42	0.92 ± 0.79	>1 year	62	0.95^a^ ± 0.85

CAT (UI/mg protein)	Men	43	1.11 ± .47	≤1 year	23	1.16 ± 0.63
	Women	42	1.24 ± .63	>1 year	62	1.18 ± 0.53

SOD (UI/mg protein)	Men	43	0.76 ± .56	≤1 year	23	0.80 ± 0.86
	Women	42	1.03 ± 1.09	>1 year	62	0.93 ± 0.88

GsH (*μ*M)	Men	43	4.42 ± 2.16	≤1 year	23	4.82 ± 2.77
	Women	42	4.03 ± 1.72	>1 year	62	4.00 ± 1.52

GPx	Men	43	0.51 ± .27	≤1 year	23	0.44 ± 0.20
	Women	42	0.65 ± 1.01	>1 year	62	0.63 ± 0.85

FRAP (*μ*M)	Men	43	278.04 ± 81.28	≤1 year	23	255.16 ± 101.75
	Women	42	233.32^a^±67.06	>1 year	62	256.24 ± 67.33

Results are expressed as mean ± SD. Values subscripted with different letters are significantly different in the same column *p* < 0.05. MDA = malondialdehyde; CAT = catalase, GPx = glutathione peroxidase, GsH = reduced glutathione, SOD = superoxide dismutase, FRAP = ferric reducing antioxidant power, LDL-C = low-density lipoprotein, TC = total cholesterol, TG = triglyceride, and HDL-C = high-density lipoprotein.

**Table 6:** Influence of folic acid intake on some markers of oxidative stress and lipid profile.

	Folic acid consumption	N	Mean ± SD	*p* value	Lipid profile	Folic acid consumption	N	Mean ± SD	*p* value
MDA (*μ*M)	No	17	1.25 ± 0.71	0.018	TC (mg/dl)	No	17	142.68 ± 41.48	0.83
	Yes	67	0.66 ± 1.39			Yes	67	145.04 ± 42.02	

CAT (UI/mg protein)	No	17	1.16 ± 0.40	0.91	TG (mg/dL)	No	17	141.47 ± 77.08	0.04
	Yes	67	1.17 ± 0.59			Yes	67	108.39 ± 53.46	

SOD (UI/mg protein)	No	17	0.76 ± 0.45	0.37	LDL-C (mg/dL)	No	17	74.09 ± 38.64	0.59
	Yes	67	0.90 ± 0.93			Yes	67	79.76 ± 41.29	

GsH (*μ*M)	No	17	3.76 ± 1.50	0.16	HDL-C (mg/dL)	No	17	40.21 ± 12.48	0.24
	Yes	67	4.38 ± 2.03			Yes	67	44.21 ± 12.25	

GPx (UI/mg protein)	No	17	0.49 ± 0.24	0.36	AIP	No		0.2197 ± 0.01	0.009
	Yes	67	0.60 ± 0.82			Yes		0.0970 ± 0.00	

Results are expressed as mean ± SD. *p* < 0.05 is statistically significant. MDA = malondialdehyde, CAT= catalase, GPx = glutathione peroxidase, GsH = reduced glutathione, SOD= superoxide dismutase, FRAP = ferric reducing antioxidant power, LDL-C = low-density lipoprotein, TC = total cholesterol, TG = triglyceride, HDL-C = high-density lipoprotein, and AIP =  atherogenic index of plasma.

**Table 7:** Influence of folic acid intake on some AIP, gender, and painful crises.

		Folic acid			*p*	Men		Women	
			No	Yes		Yes	No	Yes	No
AIP	*Low*	% within folic acid	35.3%	66.%	**0.003**	37.5%	60%	33.3%	72.2%
	*Moderate*	% within folic acid	11.8%	19.1%		0	20%	22.2%	18.2%
	*Higher*	% within folic acid	52.9%	14.7%		62.5%	20%	44.4%	9.1%

Painful crises		Yes	54.4%	45.6%		**p=0** **.041**		
		No	41.2%	58.8%	
		**p=0** **.027**		

*Risk estimated*									
*For cohort crisis = yes*	0.77								
*For cohort crisis = no*	1.320								

AIP: atherogenic plasmatic index.

**Table 8:** Correlation between lipid profile and some markers of stress.

	MDA (*μ*M)	SOD (UI/mg protein)	GsH (*μ*M)	FRAP (*μ*M)	TC (mg/dl)	TG (mg/dl)
SOD (UI/mg protein)	−0.169	1				
GsH (*μ*M)	0.579^*∗∗*^	−0.302^*∗∗*^	1			
FRAP (*μ*M)	0.588^*∗∗*^	−0.317^*∗∗*^	0.570^*∗∗*^	1		
TC (mg/dl)	−0.150	−027	−0.287^*∗∗*^	−0.299^*∗∗*^	1	
TG (mg/dl)	0.123	−0.124	0.230^*∗*^	0.226^*∗*^	0.017	1
LDL-C (mg/dl)	−0.192	0.033	−0.352^*∗∗*^	−0.335^*∗∗*^	0.913^*∗∗*^	−0.257^*∗*^
HDL-C (mg/dl)	−0.011	−0.013	−0.013	−0.120	0.329^*∗∗*^	−0.092

^*∗*^ *p* < 0.05, ^*∗∗*^*p* < 0.01; MDA = malondialdehyde; CAT = catalase; GPx = glutathione peroxidase; GsH = reduced glutathione; SOD = superoxide dismutase; FRAP = ferric reducing antioxidant power; LDL-C = low-density lipoprotein; TC = total cholesterol; TG = triglyceride; HDL-C = high-density lipoprotein.

**Table 9:** Prediction of the crisis state considering antioxidant and lipid parameters in the final model logistic regression.

		B	Wald	*p* value
Step 1^a^	CAT	−0.944	4.232	0.040
	Constant	1.027	3.257	0.071

Step 2^b^	CAT	−0.991	4.500	0.034
	LDL-C	0.014	4.021	0.045
	Constant	0.004	0.000	0.996

^a^ Variable (*s*) entered on step 1: CAT (catalase); ^b^ Variable (*s*) entered on step 2: LDL-C (low-density lipoprotein cholesterol); Wald: Wald statistic coefficient. B : the meaning of the coefficient B indicates the direction of the relationship. Thus, the relationship is positive for the LDL variable, which means that high LDL values predict the presence of crisis. On the other hand, the relationship is negative with CAT, which means that the higher the CAT values, the less likely the patient will develop a crisis.

## Data Availability

The datasets generated and analyzed during the current study can be obtained from the Laboratory of Biochemistry, Department of Biochemistry, Faculty of Medicine and Biomedical Sciences, University of Yaounde I, Cameroon.

## Abbreviations

CAT: Catalase
FRAP: Ferric reducing antioxidant power
HDL*-C*: High-density lipoprotein
GPx: Glutathione peroxidase
GsH: Reduced glutathione
LDL*-C*: Low-density lipoprotein
MDA: Malondialdehyde
SCA: Sickle cell anemia
SOD: Superoxide dismutase
*TC*: Total cholesterol
TG: Triglyceride.
